# Recent research advances in pain mechanisms in McCune–Albright syndrome thinking about the pain mechanism of FD/MAS

**DOI:** 10.1186/s13018-024-04687-y

**Published:** 2024-03-21

**Authors:** Yong Wang, Tao Jiang

**Affiliations:** Orthopedics Department, Changzhou Traditional Chinese Medicine Hospital, Nanjing University of Chinese Medicine, Changzhou, 213000 Jangsu Province China

**Keywords:** McCune–Albright syndrome, Bone remodeling, G protein-coupled receptors, GDNF family receptors, Purinergic receptors, Glycogen synthase kinase

## Abstract

**Background:**

The lack of effective understanding of the pain mechanism of McCune–Albright syndrome (MAS) has made the treatment of pain in this disease a difficult clinical challenge, and new therapeutic targets are urgently needed to address this dilemma.

**Objective:**

This paper summarizes the novel mechanisms, targets, and treatments that may produce pain in MAS and fibrous dysplasia (polyfibrous dysplasia, or FD).

**Methods:**

We conducted a systematic search in the PubMed database, Web of Science, China Knowledge Network (CNKI) with the following keywords: “McCune–Albright syndrome (MAS); polyfibrous dysplasia (FD); bone pain; bone remodeling; G protein coupled receptors; GDNF family receptors; purinergic receptors and glycogen synthase kinase”, as well as other keywords were systematically searched. Papers published between January 2018 and May 2023 were selected for finding. Initial screening was performed by reading the titles and abstracts, and available literature was screened against the inclusion and exclusion criteria.

**Results:**

In this review, we systematically analyzed the cutting-edge advances in this disease, synthesized the findings, and discussed the differences. With regard to the complete mechanistic understanding of the pain condition in FD/MAS, in particular, we collated new findings on new pathways, neurotrophic factor receptors, purinergic receptors, interferon-stimulating factors, potassium channels, protein kinases, and corresponding hormonal modulation and their respective strengths and weaknesses.

**Conclusion:**

This paper focuses on basic research to explore FD/MAS pain mechanisms. New nonneuronal and molecular mechanisms, mechanically loaded responsive neurons, and new targets for potential clinical interventions are future research directions, and a large number of animal experiments, tissue engineering techniques, and clinical trials are still needed to verify the effectiveness of the targets in the future.

## Introduction

McCune–Albright syndrome (MAS) is a genetic disorder with multiple systemic involvement, and it most often occurs in women. It is characterized by atypical symptoms, multiple fibrous dysplasia, cutaneous café au lait, and multiple endocrine disorders that can involve various organs and tissues, including the gonads, thyroid, pituitary, and adrenal glands. The disease has a prevalence of 1 in 100, 000 to 1 in a million and exists in both mono- and poly-osteogenic forms. The disease is characterized by the formation of cranial skeletal dysplasia or mesial-extremity skeletal lesions with intertwining fibrous tissue with normal bone, as well as the accumulation of nonmineralized bone and increased osteoclast activity. Somatic mutations resulting from activating mutations in the gene (GNAS1) encoding the α-subunit of the guanine nucleotide-binding protein (Gsα) during early embryonic life are the main cause of the disease. Because of the mosaic distribution of mutations, it results in a low rate of positive genetic tests leading to adenylate cyclase stimulation and cyclic AMP production [1], as well as the proliferation of bone progenitor cells and the lesion of poly-fibrous dysplasia (FD).

The disease has been reviewed by Tseng [2] and Jiang [3]; however, they only focused on the statement of existing symptoms and the summary of diagnostic methods, the most serious pain mechanism of this disease has not been discussed. The focus of this article was to review the pain mechanisms of FD/MAS in axial limb skeletal lesions. This disease can lead to bone pain, spinal deformities, and fractures; moreover, corrective surgery, although commonly used in FD/MAS, does not provide sustained pain relief. Currently, surgical procedures and existing drugs that alter bone turnover pathways also have obvious drawbacks, and the incomplete correlation between disease lesion load and patient-reported pain intensity adds to the complexity of FD/MAS pain management [4]. Due to the lack of effective international understanding of the pain mechanisms in this disease, the pain treatment of FD/MAS remains a difficult problem to overcome in clinical practice. Therefore, this paper reviews recent research on pain mechanisms in FD/MAS, such as mechanosensitive channels, redox, acidosis, specific hormone-regulated abnormal bone remodeling, signaling pathways regulating pain, interferon-stimulated factors, and key receptors; in addition, it describes new approaches to treat pain in MAS to explore new therapeutic targets for pain in this disease, as well as tissue engineering approaches.

## Materials and methods

We performed a database search on PubMed, Web of Science, China Knowledge Network (CNKI) and selected papers that were published between January 2018 and May 2023 in the English language. PubMed was last accessed on May 30th, 2023. The following keywords and terms were used: (1) McCune–Albright syndrome (MAS); (2) poly-fibrous displasia (FD); (3) bone pain; (4) bone remodeling; (5) G protein coupled receptors; (6) GDNF family receptors; (7) purinergic receptors; and (8) glycogen synthase kinase. The following string was used:

(((((((("Fibrous Dysplasia, Polyostotic" [Mesh] or Dysplasia, Polyostotic Fibrous or Dysplasias, Polyostotic Fibrous or Fibrous Dysplasias, Polyostotic or Polyostotic Fibrous Dysplasias or Polyostotic Fibrous Dysplasia or Albright Syndrome or Syndrome, Albright or McCune–Albright Syndrome or McCune Albright Syndrome or Syndrome, McCune–Albright or Albright’s Disease or Fibrous Dysplasia with Pigmentary Skin Changes and Precocious Puberty or Albright’s Syndrome with Precocious Puberty or Albright-Mccune-Sternberg Syndrome or Albright-Sternberg Syndrome or Albright’s Syndrome or Syndrome, Albright’s or Albright’s Disease of Bone) OR ("Bone Remodeling" [Mesh] or Remodeling, Bone or Bone Turnover or Bone Turnovers or Turnover, Bone or Turnovers, Bone and pain)) OR ("Receptors, G-Protein-Coupled" [Mesh] or Receptors, G Protein Coupled or G-Protein-Coupled Receptors or G Protein Coupled Receptors or G-Protein-Coupled Receptor or Receptor, G-Protein-Coupled or G Protein Coupled Receptor and pain)) OR ("Glial Cell Line-Derived Neurotrophic Factor Receptors" [Mesh] or Glial Cell Line Derived Neurotrophic Factor Receptors or GDNF Receptors or GFRA4 Receptor or GDNF Family Receptor Alpha 4 or Glial Derived Neurotrophic Factor Receptor 4 or GDNF Family Receptor 4 or GFRA3 Receptor or GDNF Family Receptor Alpha 3 or Glial Derived Neurotrophic Factor Receptor 3 or GDNF Family Receptor 3 or GFRA1 Receptor or GDNF Family Receptor 1 or GDNF Family Receptor Alpha 1 or Glial Derived Neurotrophic Factor Receptor 1 or GFRA2 Receptor or GDNF Family Receptor Alpha 2 or Glial Derived Neurotrophic Factor Receptor 2 or GDNF Family Receptor 2 and pain)) OR ("Receptors, Purinergic" [Mesh] or Purine Receptor or Receptor, Purine or Purinoceptors or Purine Receptors or Purinergic Receptors or Purinoceptor or Purinergic Receptor or Receptor, Purinergic or Receptors, Purine or Methyladenine Receptors or Receptors, Methyladenine and pain)) OR ("Glycogen Synthase Kinases" [Mesh] or Kinases, Glycogen Synthase or Glycogen Synthase Kinase or Kinase, Glycogen Synthase or Synthase Kinase, Glycogen and pain)) OR ("Bites and Stings" [Mesh] or Stings and Bites or Stings or Sting or Bites or Bite and pain)) OR ("P2RX7 protein, human" [Supplementary Concept] and bone pain)) OR ("Cyclic GMP-Dependent Protein Kinases" [Mesh] and pain).

In addition, a manual search was performed of the cited references in the included studies. The reviewers (YW and TJ) retrieved the data and independently analyzed each selected study; instances of disagreement were resolved by the senior investigator (TJ).

A total of 1, 778 papers were identified with no duplicates; additionally, as a first step, 110 papers were excluded for other reasons, and 122 papers were excluded due to duplication (PRISMA flow diagram reported in Fig. 2). A total of 712 articles that did not meet the requirements by reading the abstracts and titles were excluded. However, there were 78 unresearched literature reports, which resulted in 988 papers for further evaluation. According to the selection criteria, out of the 988 qualified research results that were evaluated by PubMed, 15 articles were ultimately included in this analysis.

## Results

### Mechanisms by which abnormal bone remodeling affects FD/MAS pain

#### Mechanically sensitive channel Piezo family

FD/MAS is a genetic skeletal disorder that is characterized by abnormal bone remodeling, and studies have found that bone remodeling and inflammatory processes are thought to produce bone pain in FD/MAS [5]. The Piezo family includes Piezo1 and Piezo2 subtypes, and the most relevant stressor regulating Piezo1 is mechanical force, which is a key factor in bone remodeling. Piezo1 ion channel mediatesosteoblasts’ mechanical force sensitivity and its important role is load-dependent bone formation, suggesting a new mechanism for mechanical force sensing in bone tissue. When sensory nerve fibers swell in response to mechanical stress, they cause the activation of mechanosensitive ion channels, which are recruited in osteoblasts and chondrocytes, thereby regulating osteogenesis and cartilage degradation in joints and promoting the process of bone remodeling [6]. Piezo1 is detected in osteoblasts (which are the most abundant bone cells), and Piezo1-Akt channels are key players in tissue remodeling, wherein they mainly act on osteoblasts. Among these signaling effects, Piezo1-Akt signaling and the inhibition of sclerostin (which is an important regulator of bone formation), as well as its downregulation, affect osteoblast function in FD/MAS patients, and the activation of the Piezo1-Akt pathway in osteoblasts is needed for mechanical stretch-induced downregulation of sclerostin (Sost) expression. Therefore, Piezo1 was identified as a major skeletal mechanosensor regulating skeletal homeostasis, which raises the possibility for new selective therapeutic approaches for FD/MAS [7, 8].

FD/MAS generates intraosseous pressure in the bone microenvironment, whereby it sensitizes primary afferent nerves through the activation of mechanoreceptors and osteocyte mechanotransduction. Piezo2 has been found to be critical for transferring pain and touch sensation, as well as proprioception, in the nervous system, and irreversible in vivo Piezo2 microdamage may be an important contributor to FD/MAS pain [9]. It has been suggested that primary Piezo2 injury is the primary pain pathway in FD/MAS because of the loss of unbalanced subthreshold calcium currents and N-methyl-D-aspartate (NMDA) activation, as well as due to the dysregulation of the primary pain pathway in the dorsal horn of the spinal cord (due to NMDA activation) and the loss of l-type calcium currents, thus resulting in the loss of activation of wide dynamic range neurons. Animal studies have demonstrated that loss-of-function mutations in Piezo2 lead to loss of pain perception, whereas irreversible Piezo2 microdamage can continuously activate the transcriptional pathway [10].

Additional studies have suggested that Piezo2 plays a role in synchronizing supraspinal neural networks [11], and the loss of excitatory function of Piezo2 may theoretically lead to impaired spinal synchrony and loss of spinal function in the central pattern generator (CPG). Pathogenic variants of Cav1.3, which encode the CACNA1D gene, may play a role in the pain mechanisms of FD/MAS and have a specific relationship with dysregulation of pain pathways in the dorsal horn of the spinal cord [12]. Table 1 summarizes studies on the mechanisms by which the Piezo family affects pain through abnormal bone remodeling.

**Table 1:** Summary of studies on the mechanisms by which the Piezo family affects pain through abnormal bone remodeling

Year	Author (with literature)	Research methodology	Main results	Main conclusions
2018	Szczot et al. [10]	Mouse animal experiments	Piezo2 can lead to loss of activation of a wide dynamic range of neurons. Simultaneous irreversible Piezo2 microdamage can continuously activate transcriptional pathways	Primary damage to Piezo2 may be one of the main pain targets in FD/MAS
2019	Sasaki et al. [8]	Osteoblast experiment	Piezo1-Akt channels are key players in mechanosensitivity and subsequent tissue remodeling, primarily by acting on osteoblasts	Piezo1 was identified as the primary skeletal mechanosensor regulating skeletal homeostasis in FD/MAS pain mechanisms, and they play a key role
2020	Wang et al. [7]	Mouse animal experiments	Piezo2 can affect osteoblast-osteoclast crosstalk in response to mechanical forces. In response to mechanical loading, Piezo1 in osteoblasts controls the YAP-dependent expression of type II and type IX collagen1	Piezo2 was identified as the primary skeletal mechanosensor, thereby affecting bone remodeling. It plays a key role in FD/MAS pain mechanisms
2021	Hendrickx G et al. [6]	Mouse animal experiments	Evidence that Runx2② is expressed not only in osteoblast progenitor cells but also in hypertrophic prechondrocytes suggests that Piezo1 plays a role in chondrocytes to ensure bone trabecule formation	Regulates osteogenesis and cartilage degradation in the joint and promotes the process of bone remodeling
2023	Perin et al. [13]	Overview	Triggers osteoblast differentiation and hinders lipogenic cell differentiation	This mechanism affects bone remodeling. It may play a key role in FD/MAS pain mechanisms
2023	Sonkodi et al. [9]	Mouse animal experiments	Piezo2 is essential for shifting pain and touch sensations, as well as proprioception, in the nervous system	Irreversible microdamage of Piezo2 ion channels may be a major cause of MAS pain
2023	Nagy et al. [11]	Genome Analysis	The absence of pathogenic variants of Piezo2 indicates a new theory of noncontact damage mechanisms	Variants in Cav1.3 encoding the CACNA1D gene (iii) may be responsible for MAS bone pain

① YAP-dependent expression: Yes-associated protein (YAP for short), which preferentially uses fatty acid oxidation for energy supply; ② Runt-related transcription Factor 2: Runx2 plays an important role in the regulation of skeletal-related genes, which regulate the process of bone resorption, bone formation, and bone reconstruction ( in addition to regulating osteoblasts, chondrocytes, osteoclasts, and other bone cells); ③ CACNA1D is a voltage-gated calcium channel

#### Role of redox homeostasis in abnormal bone remodeling pain

Oxidative stress (OS) is a potential injury resulting from the disruption of the balance of strong oxidants and antioxidants, which plays an important role in the physiopathological and aging processes of many tissues. Its broad role is in bone tissue, where reactive oxygen species (ROS) are involved in bone remodeling via the RANKL pathway, thus physiologically promoting osteoclast differentiation [14]. The redox state should be kept in a precise balance to maintain proper bone remodeling; however, due to increased osteoclast activity, FD/MAS predisposes to ROS accumulation and cellular stress; additionally, ROS promote osteoblast apoptosis and indirectly elicit osteoclast formation, and excessive mechanical forces not only affect bone mass and its microarchitecture but also promote inflammatory responses through redox affecting NF-κB factors. Thus, redox dysregulation affecting bone remodeling plays an important role in the pathogenesis of FD/MAS pain [14]. Epithelial sodium channels (ENaCs) are major contributors to intracellular sodium homeostasis. Specifically, αENaC subunits are present in skeletal cells, including articular chondrocytes and osteoblasts, and are thought to be involved in mechanotransduction, sodium transport, and extracellular sodium sensing. It was found that there is a correlation between bone sodium content and FD/MAS, as well as the fact that ENaC is sensitive not only to extracellular sodium and mechanical forces but also to oxidative stress. Moreover, it has been shown that ENaC is upregulated through ROS production and that a similar correlation between ENaC function or expression and ROS production in osteoblasts may provide a new research direction for FD/MAS [15].

### The role of acidosis in the pain mechanism of FD/mas bone remodeling

#### Acid-sensing ion channel (ASIC)

Acid-sensing ion channels (ASICs) are a group of proton-gated cation-permeable channels, and reduced osteoblastogenesis was observed under acidic conditions when osteoblasts were cultured in medium with elevated pH, whereas ASIC2, ASIC3, and ASIC4 were progressively upregulated. The authors suggested that decreased pH activates acid-sensing ion channels and that acidic pH leads to impaired osteoblastogenesis, which represents a pH-dependent pattern that may support a role for ASIC in the painful mechanism of FD/MAS bone remodeling [16].

#### Transient receptor potential (TRP) family channels

Transient receptor potential (TRP) channels, which are a class of cation channels, are widely expressed cation channels that play an important role in mediating calcium homeostasis and are considered to be potential regulators of inflammatory pain. Skeletal disorders with increased bone resorption by osteoclasts are usually associated with pain, and they secrete large amounts of protons during bone resorption, thus resulting in an acidic local environment. Acidosis is a typical noxious stimulus that innervates injured skeletal nerves and can excite injurious sensory neurons by opening acid-sensing ion channels (ASIC) and transient receptor potential channels [17]. The transient receptor potential channel subfamily V (TRPV) channels TRPV1, TRPA1, and TRPV4, as well as the transient receptor potential cation channel subfamily M (TRPM7) channels, have been reported to be present in bone; however, only TRPV1 and TRPA1 respond to pH changes [18]. However, in a mildly acidotic environment, OC formation is also increased when the specific agonist 4-alpha PDD activates TRPV4, thus indirectly causing acidosis and increasing FD/MAS pain risk [19].

Altogether, TRPV4, TRPV1, and TRPV2 are involved in acidosis, as well as in promoting osteoclast formation and impairing bone formation by enhancing calcium ion excretion and osteoclast resorption. TRPA1 is distributed in a large number of cell types and is usually associated with TRPV1, which is sensitive to external stimuli (especially in nerve tissue). Tissue stiffness in FD/MAS patients activates TRPV4 and increases M1 macrophage infiltration, and TRPV1 has a prominent role with TRPV4 in external stimuli and inflammatory environmental episodes; thus, this ion channel is considered a potential therapeutic target for FD/MAS pain. TRPM7 is a TRP channel involved in bone metabolism, as well as a cation channel that is covalently linked to the structural domain of protein kinase; additionally, it has been found that TRPM7 acts as a cation channel in bone. TRPM7 has also been found to play a key role in bone metabolism as a cation channel.

In the development of FD/MAS, formaldehyde can be secreted by disease-involved tissues. In pain models, formaldehyde was found to upregulate TRPV1 expression and contribute to pain behavior. These facts suggest that the targeting of formaldehyde production may be a potential therapeutic approach for FD/MAS pain. A summary of studies on the mechanisms by which transient receptor potential channels (TRPs) influence abnormal bone remodeling is detailed in Table 2.

**Table 2:** Summary of studies on the mechanism of transient receptor potential channels (TRP) affecting abnormal bone remodeling

Year	Author (with literature)	Research methodology	Main results	Main conclusions
2015	Liu et al. [20]	Cellular experiments	Osteogenesis of bone marrow stromal cells can be promoted by low intensity pulsed ultrasound/nanomechanical force generator by modulating TRPM7 ①, actin cytoskeleton, and intracellular calcium oscillations	TRPM7 can affect bone remodeling and plays an important role in regulating the process of joint osteogenesis
2018	Son, et al. [21]	Mouse animal experiments	In mouse osteoblasts, low osmosis increases intracellular calcium via TRPV4② and TRPM3 and regulates the expression of RANKL③ and NFATc1④	This study could demonstrate that TRP ⑤channels activated by mechanical stress may play a key role in bone remodeling in MAS
2018	Corrigan, et al. [22]	Cellular experiments	TRPV4 is preferentially localized to MSC osteoblast precursors and differentiated osteocytes	It has been demonstrated that TRPV4 is needed for MSC mechanotransduction, thus mediating oscillatory fluid shear-induced calcium signaling and early osteogenic gene expression
2022	Du et al. [23]	Cellular experiments	TRPV4-mediated Ca^2+^ signaling plays a central role in the chondrocyte response to 197 kPa and 78 kPa substrates, whereas Piezo1/2-mediated Ca^2+^ signaling plays a central role in the chondrocyte response to 54 kPa and 2 kPa substrates	TRPV4 can exacerbate bone pain in MAS by affecting chondrocytes
2020	Dutta, et al. [24]	Cellular experiments	Hypotonic conditions trigger ROS3 ⑥production by TRPV4 in synovial cells, and tissue sclerosis activates TRPV4 and increases M1 macrophage infiltration	The inflammatory response that is present in MAS may exacerbate pain due to hypoosmotic conditions by increasing TRPV4 in synovial cells
2023	Perin et al. [13]	Cellular experiments	Increased OC formation was observed after activation of TRPV4 by the specific agonist 4-alpha PDD in a mildly acidotic environment	Activation of TRPV4 has a facilitative effect on OC formation, thus proving to play an important role in bone remodeling in MAS
2020	Liu et al. [25]	Animal experiments on rats	SHP-1 in sensory neurons is an endogenous analgesic and delays the development of bone cancer pain by inhibiting TRPV1 function	In the early stages of MAS, patients feel no pain or mild pain, which is likely due to the anti-injury sensing effect of programmed death ligand 1 (PD-L1)
2022	Wen Chen etc. [26]	Overview	The role and potential mechanisms of TRPV1 in DRG⑦ for bone pain are reviewed	The study found that formaldehyde upregulated TRPV1 expression and contributed to alleviating MAS pain behavior

① transient receptor potential channel subfamily V (TRPV) channel; ② transient receptor potential cation channel subfamily M (TRPM7) channel; ③ RANKL: nuclear factor κ B receptor activator ligand; ④ NFATc1: NFATc1 is thought to be a key gene in the musculoskeletal system that regulates osteoclast differentiation and function; ⑤ transient receptor potential channels (TRP); ⑥ reactive oxygen species (ROS) is a highly reactive chemical containing oxygen radicals; ⑦ dorsal root ganglia (DRG)

### Summary of hormones associated with painful abnormal bone remodeling

Current research suggests that abnormal bone growth in FD/MAS leads to structural and functional alterations in nerve fibers and increased sensitivity to pain. The overproduction of hormones acting on nociceptive nerves can also lead to pain.

#### Role of parathyroid hormone in abnormal bone remodeling

Parathyroid hormone (PTH) regulates the body’s calcium homeostasis. In vitro and in vivo studies have shown that PTH directly activates survival signaling in osteoblasts, thus leading to an increase in the number of osteoblasts through a delay in osteoblast apoptosis, which correspondingly affects abnormal bone remodeling [27]. In a rat model of PD (Parkinson’s disease), PTH was found to have anti-inflammatory and inhibitory effects on bone loss. Cortisol inhibits parathyroid hormone and stimulates the proliferation and differentiation of progenitor cells into osteoclasts. Administration of cortisol may attenuate bone remodeling in patients with MAS by inhibiting bone formation [28]. Therefore, cortisol may improve MAS pain as a therapy, but not optimally.

#### Oxytocin regulates bone formation

Oxytocin (OT) is produced by the hypothalamus, and osteoblasts expressing OT receptors can bind to it. It was found that OTs are involved in the bone remodeling process, which reduces bone resorption and leads to a relative increase in bone formation. The OT treatment process leads to increased intracellular calcium levels and modulates the stimulation of osteoblast formation, thus regulating bone formation [29].

#### Insulin is involved in activating abnormal bone remodeling

Insulin has been reported to activate osteoblast differentiation, reduce apoptosis of osteoblasts, and decrease osteoclast activity. Insulin-like growth factor-1 (IGF-1) can be activated by human growth hormone and can also be secreted by osteoblasts. IGF-1 is involved in abnormal bone remodeling during FD/MAS, and IGF-1 synthesis is regulated by parathyroid hormone (PTH) [30]. A low decrease in IGF-1 expression is thought to be needed for the onset of apoptosis and to promote osteoblast differentiation.

#### Sex hormones are involved in activating abnormal bone remodeling

Estrogen (ES) plays an important role in the regulation of skeletal maturation and bone remodeling. At the cellular level, ES inhibits osteoclast differentiation, and deficiency in ES leads to increased osteoclast formation, which reduces osteoclast number and decreases the number of active remodeling units [31]. Testosterone activates osteoblasts by activating steroid receptors (either directly or via aromatization to estradiol) to regulate FD/MAS abnormal bone remodeling [32].

#### Erythropoietin is involved in abnormal bone remodeling

Erythropoietin (EPO) can indirectly increase bone formation by increasing the expression of vascular endothelial growth factor (VEGF), and EPO usually indirectly activates osteoblast differentiation. These empirical findings are important for understanding bone remodeling and may be useful in treating bone defect growth issues [33]. EPO can promote bone formation directly and indirectly through VEGF.

#### Abnormal bone remodeling involving lipocalin

Lipocalin (AN) is a protein produced by adipose tissue, and studies have identified a potential role for AN as a positive bone mass regulator, angiogenesis stimulator, and osteoclast suppressor. Two AN receptors have been identified by the expression of osteoblasts, thus suggesting a role for AN in bone metabolism. Additionally, it has a direct function in bone metabolism by promoting proliferation and stimulating bone formation, as well as possessing the potential for bone regeneration [34]. AN and its agonists may be used to treat FD/MAS abnormal bone remodeling pain.

## Nociceptive sensitization and the role of G protein-coupled receptors (GPCRs) in FD/MAS pain

In FD/MAS, the articular cartilage is not innervated and does not become a direct source of pain, whereas the rest of the tissue has nociceptive receptors and sympathetic nerve fibers. Nociceptive receptors that are continuously stimulated respond to harmless painful stimuli or respond strongly to harmful pain, which is a phenomenon known as nociceptive sensitization [35]. The spinal dorsal horn is the first transit point for pain signals, which are transmitted to the dorsal horn of the spinal cord and subsequently to the upper brain centers, thus creating conscious pain. Numerous studies have shown that considerable amounts of pain are mediated by both central and peripheral sensitization. Peripheral sensitization leads to abnormal hyperexcitability of primary sensory neurons, whereas central sensitization enhances injurious information from the dorsal horn of the spinal cord to the cerebral cortex, in regards to synaptic transmission [36].

G protein-coupled receptors (GPCRs) can sometimes indirectly sensitize various voltage-gated ion channels (VGICs) that are expressed on sensory neurons, thereby further enhancing nociceptive signals to the spinal cord. Studies have demonstrated that ovarian cancer G protein-coupled receptor 1 (OGR1) family subtypes are differentially expressed in different inflammatory pain states and that they are involved in short- and long-term chronic inflammatory pain. Alkaline pH promotes mineralization and osteoblast potential, whereas acidosis correspondingly stimulates osteoclast resorption. OC sensitivity to protons involves the early expression of ovarian cancer G protein-coupled receptor 1 (OGR1), and studies have suggested that OGR1 is involved in osteoclast formation and that OGR1 may be a central acid sensor in bone**,** which is important for studying FD/MAS-induced pain mechanisms [37].

## Dual role of the NO/cGMP (cyclic guanosine monophosphate) signaling pathway

The main process of the NO/cGMP signaling pathway in cells involves NO activation of soluble guanylate cyclase, which leads to subsequent production of cGMP. The activation of NO/cGMP signaling in the spinal cord significantly induces upregulation of downstream effectors, as well as reactive astrogliosis and microglia polarization involved in the chronic pain process. In dorsal root ganglion neurons, natriuretic peptides bind to granular guanylate cyclase to produce and further activate the cGMP/PKG pathway, thus contributing to the development of FD/MAS pain. In addition, FD/MAS leads to the upregulation of multiple receptors that are involved in the activation of the NO/cGMP signaling pathway in various types of pain, and the NO/cGMP signaling pathway induces the expression of downstream effectors that exert analgesic effects in both neuropathic and inflammatory pain [38].

Lactoferrin can exert its antinociceptive sensitization effects by activating the NO-cGMP-ATP-sensitive K-channel signaling pathway, and this interaction is used in inflammatory and injurious pain treatment. The activation of NO/cGMP signaling was found to play an important role in the development of chronic pain, and this signaling pathway with dual roles is a promising target for FD/MAS pain treatment [39].

## Effects of the ERK/CREB pathway on FD/MAS pain mechanisms

Extracellularly regulated protein kinases (ERKs) play important roles as cell signaling transducers in a variety of essential cellular functions, such as migration, differentiation, growth, and survival, whereas cyclic phospho-adenosine effector-binding proteins (CREBs) regulate the transcription of a variety of cellular genes, including dopaminergic neurons. Moreover, the activation of CREBs can improve muscle performance. It has been shown that recombinant Sirtuin 6 (SIRT6) downregulates Sox6 (which is a key blocker of slow fiber-specific genes) by increasing CREB transcription, which improves muscle performance; this effect represents another new direction in the study of FD/MAS pain mechanisms [40].

### Oxidative stress and neuroinflammation

The BDNF-TrkB-ERK-CREB pathway is extensively involved in neurologically relevant processes, and the BDNF-TrkB-ERK-CREB pathway is closely associated with oxidative stress and neuroinflammatory processes. Ectopic ossification in FD/MAS patients can detect elevated sensory innervation, and activated sensory fibers can release neuropeptide P and calcitonin gene-related peptide (CGRP) via the induction of neuroinflammation [41]. It has been demonstrated that increased pain and pain aversion in bone pain rats is accompanied by the upregulation of brain-derived neurotrophic factor (BDNF) expression, and the phosphorylation of the ERK/CREB pathway (pERK/pCREB) is upregulated in response to increased pain and pain aversion (or the exogenous injection of BDNF). The decreased expression level of pERK/pCREB after blocking BDNF-TrkB signaling suggests that the ERK/CREB pathway is critical for BDNF-TrkB signaling-mediated pain and aversion pain, and this ERK/CREB pathway may induce pain in FD/MAS by mediating oxidative stress and neuroinflammatory processes [42].

### miRNA-mediated activation of the ERK/CREB pathway

During pain, the activation of the ERK/CREB pathway is often mediated by microRNAs. In chronic inflammatory rats, miR-211 can target the 3’-UTR of the ERK gene, which greatly increases the expression levels of ERK and CREB, thus promoting hypersensitivity and consequently pain [43]. MicroRNA-365 decreases the expression of β-arrestin2, ERK, and CREB proteins, and β-arrestin2 negatively targets and inhibits ERK/CREB pathway activation to achieve better analgesic effects [44].

## Modulation of FD/MAS pain by purinergic receptors (P2XRs)

Purinergic receptors (P2XRs) are ligand-gated cation channels that are mainly activated by extracellular ATP (eATP). Some P2XRs are found in osteoclast, and P2XR members regulate bone resorption, particularly through the proinflammatory microenvironment [45]. P2X3R was found to be distributed not only in the peripheral nervous system but also in the presynaptic portion of the spinal cord and in the periaqueductal gray matter (PAG) of the midbrain. Tian Shuxin et al. showed that both peripheral and central P2X3Rs are involved in analgesia [46]. Moreover, Liu Min reported that the functional upregulation of P2X3R is involved in the pathogenesis of pain, as well as the fact that P2X3 receptors are involved in injurious transmission and modulation in the peripheral and central nervous system and that P2X3R blocks mechanically abnormal pain and nociceptive hyperalgesia, thus exerting a significant analgesic effect [47]. P2X7R is the most abundant form of receptor in osteoclasts, and P2X7R mechanotransduction mediates not only osteoclast proliferation but also bone resorption, pain, and reduced mechanical sensitivity. The author suggests that blockage of the P2X3 receptor is a new therapeutic target for FD/MAS pain [48]. A summary of studies on purinergic receptors on pain mechanisms is detailed in Table 3.

**Table 3:** Summary of studies on purinergic receptors in pain mechanisms

Year	Author (with literature)	Research methodology	Main results	Main conclusions
2018	Falk et al. [49]	Animal experiments on rats	The P2X7R ion channel is expressed during OC (osteoclast) maturation, and it mediates inflammatory vesicle activation and IL-1β① release, which is needed for OC-mediated bone loss; it also mediates reduced mechanosensitivity of osteocytes	P2X7R may be a potential pharmacological target for the treatment of MAS and could lead us to further investigate the role of this receptor in the progression of bone remodeling pain
2023	Sun et al. [50]	Overview	P2X4 receptors mediate neuropathic pain in spinal cord injury and are significantly involved in mediating the pain hypersensitivity response to increased BDNF and p38-MAPK ②	P2X4R and P2X7R may be trigger molecules in exercise-mediated changes in BDNF③ and provide a possible mechanism for MAS pain protection
2022	Yuan et al. [47]	Animal experiments on rats	Pharmacological blockade of P2X3R has a significant analgesic effect on mechanical abnormal pain and thermal nociceptive sensitization	It may serve as an important target for blocking pain during MAS
2023	Tian et al. [46]	Animal experiments on rats	P2X3R is involved in analgesia, and P2X3R is distributed not only in the peripheral nervous system (such as primary afferent central terminals) but also in the presynaptic portion of the spinal cord and in the periaqueductal gray matter (PAG) of the midbrain conductors	MAS pain process can be treated by blocking P2X3R as an important method of pain treatment

① IL-1β: interleukin-1β is an important cytokine and peptide regulator that is mainly produced by monocytes. It plays a regulatory role in cellular immune activation; ② p38-MAPK: P38 protein kinase; ③ brain-derived neurotrophic factor (BDNF)

## Important player involving GDNF family receptors in FD/MAS pain

Glial cell-derived neurotrophic factor (GDNF) has been shown to sensitize injury receptors and induce chronic inflammatory pain and muscle pain. GFRα is a high-affinity receptor for GDNF, and the knockdown of GFRα1 by intrathecal injection of antisense oligodeoxynucleotides, as well as mRNA encoding GFRα1, attenuates both GDNF-induced nociceptive hyperalgesia and chronic muscle pain. GDNF-GFRα1-Ret-ERK signaling has been reported to activate Runx2-mediated P3X1R gene transcription, which contributes to the sensitization of DRG neurons and induces bone pain; additionally, this study may provide a potential target mechanism leading to bone pain in FD/MAS patients. Inflammatory bone pain involves the activation of the GDNF-GFRα1 signaling pathway and the sensitization of nonpeptidergic bone afferent neurons, and neuroinflammation occurring in FD/MAS patients can be induced by GDNF expression in activated astrocytes, microglia, and infiltrating macrophages [51]. Studies have shown that bone afferent neurons of GFRα1 and GFRα2 are mostly nonpeptidergic; therefore, GDNF and neuroturin signaling through nonpeptidergic sensory neurons may be important for bone pain in FD/MAS. However, GDNF isolation does not prevent inflammation-induced pain behavior and may contribute to the maintenance of deep tissue pain [52]. Artemin, which is a member of the neurotrophic factor family derived from the glial cell lineage, is an important player in persistent pain, such as neuropathic cold pain and inflammatory bone pain [53]. It has been found that inflammatory bone pain in FD/MAS involves the activation of NGF-sensitive peptidergic neurons via the artemin/GFRα3 signaling pathway [54]. Moreover, the activation of the artemin/GFRα3 pathway can directly or indirectly activate TRP channel expression and activity and perpetuate pain; thus, it is known that artemin/GFRα3 plays an important role in FD/MAS pain [55]. Table 4 provides a detailed summary of the potential targeting mechanisms of the GDNF family of receptors for bone pain.

**Table 4:** Summary of studies on potential targeting mechanisms of brain-derived neurotrophic factor (BDNF) family receptors for bone pain

Year	Author (with literature)	Research methodology	Main results	Main conclusions
2022	Messina et al. [54]	Animal experiments on rats	Inflammatory bone pain involves the activation and sensitization of nonpeptidergic neurons via the GDNF/GFRα1① and neuroturin/GFRα2 signaling pathways, and the chelation of antibodies to nerve growth factor may be useful in the treatment of inflammatory bone pain caused by the activation of GDNF-sensitive nonpeptidergic bone afferent neurons	FD/MAS patients exhibit activation of inflammatory bone pain, whereas GDNF/GFRα1 and neuroturin/GFRα2 signaling pathways activate and sensitize nonpeptidergic neurons to help treat patient pain
2022	Minnema, et al. [55]	Mouse animal experiments	Bone pain may activate and sensitize neurons through GDNF/GFRα1 and neurturin/GFRα2artemin/GFRα3② signaling pathways	The results suggest a functional role for artemin/GFRα3 in the development of FD/MAS pain
2022	O-Sullivan et al. [56]	Mouse animal experiments	Enhanced NGF/TrkA③ signaling in articular synovial and peripheral sensory neurons promotes pro-injury sensitization and focused pain sensitization	The NGF/TrkA pathway is critical for shifting changes in pain and neural processing, and FD/MAS induces the expression of the brain-derived pain mediator BDNF via peripheral NGF

① GDNF/GFRα: GFRα is a high-affinity receptor for GDNF; ② NGF/TrkA: nerve growth factor (NGF)/promyosin receptor kinase A (TrKA) signaling axis; ③ Glial cell line-derived neurotrophic factor receptor α3

## Interferon-stimulating factor (STING): a new target for FD/MAS pain

STING is an intracellular DNA sensor with an important role in chronic pain. DRG neurons are involved in interferon-stimulated factor (STING), which mediates the stimulus of the innate immune response and regulates injury perception. Moreover, upon binding to DNA, cyclic GMP-AMP synthase (cGAS) undergoes a conformational change to an active state and generates a second messenger cyclic GMP-AMP (cGAMP) [57]. cGAMP acts as a second messenger to activate STING and drives STING translocation from the endoplasmic reticulum to perinuclear microsomes via the Golgi apparatus [58]. Subsequently, activated STING recruits and phosphorylates TANK-binding kinase 1 (TBK1), which further activates interferon regulatory factor 1 (IRF1) and NF-κB factors. These factors enter into the nucleus to induce the expression of type I interferon (IFN-I) and proinflammatory factors, such as IL-6β, IL-7, and TNF-α [59]. Previous studies have demonstrated that the cGAS-STING pathway is a potential mechanism for many inflammation-mediated pathophysiological processes [60]. Although there is growing evidence that STING is involved in the generation and maintenance of neuroinflammation and inflammatory bone pain, a recent study in an alternate nerve injury (SNI) model demonstrated that STING activation in DRG neurons drives bone pain through the TBK1/IKK/NF-κB proinflammatory factor signaling pathway; thus, STING may be a new therapeutic FD/MAS pain target for the treatment of FD/MAS pain [61].

## Potassium channel involvement in patients with FD/MAS

Patients with FD/MAS may evoke hypernormal activation of neuronal resonance and disturbances in neuroplasticity through enhanced responses to sensory input. These processes may involve imbalance at the neuronal level, thus leading to hyperexcitability and hypersensitivity of neurons in the CNS, which results in sustained activation of injury receptors; these findings are consistent with the development of central sensitization [62]. K channels are involved in fibromyalgia in FD/MAS, with channels including fast K + channels, potassium leak (KL) channels, KCHN2 voltage-gated potassium channels, and Kv channel autoantibodies. Additionally, it has been found that the water fraction of substance P (SP) is elevated in the cerebrospinal fluid of FD/MAS patients [63]. Substance P is a mediator of pain, is involved in pain production and transmission signaling, and can also exhibit anti-injury-sensing properties.

## Potential new approaches to treat FD/MAS pain

Glycogen synthase kinase-3 (GSK-3) is a cytoplasmic serine/threonine protein kinase that is involved in a large number of key cellular processes, and kenpaullone (kp) is a glycogen synthase kinase-3 (GSK3)/cell cycle protein-dependent kinase (CDK) inhibitor [64]. In vitro and in vivo studies have found that kp acts as an analgesic in a preclinical mouse model of pathological pain and that its cellular mechanism of action in neurons is based on its GSK-3β inhibitory function, which enhances Kcc2/KCC2 gene expression. In turn, Kcc2 upregulation is dependent on the nuclear translocation of the neuronal connexin δ-connexin (δ-cat). The enhancement of Kcc2 gene expression by KAISO transcription factors and increased Kcc2 gene expression leads to increased KCC2 transporter proteins in neurons. Furthermore, kP exhibits a robust analgesic response in mice, and the defect in KCC2 expression in SCDH is repaired by KP [65].

**Fig. 1:**
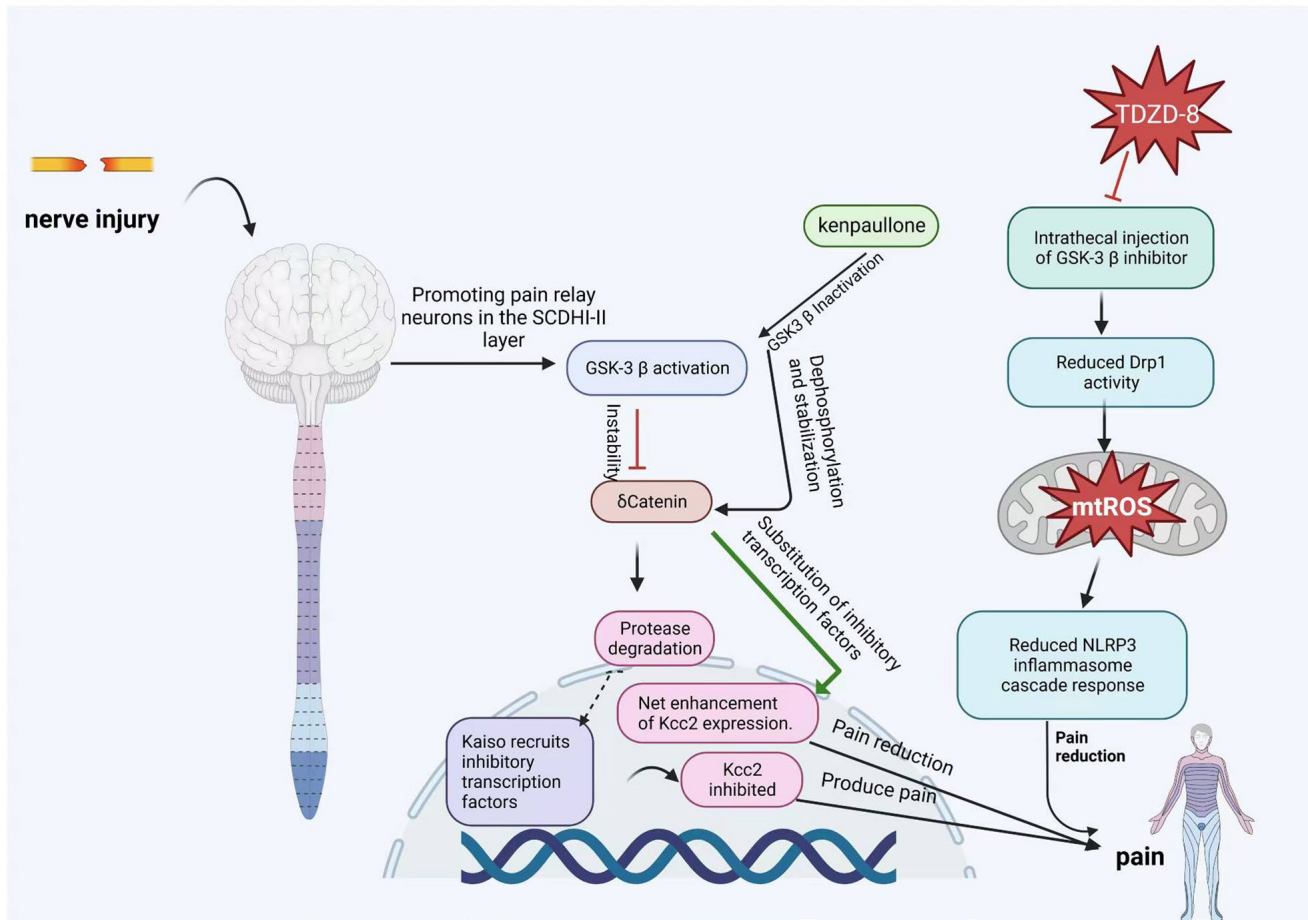
The analgesic mechanism of kenpaulone and TDZD. *Notes*: ① NLRP3 inflammasome, which is a cellular structure composed of multiple proteins, is involved in immune and inflammatory responses; ② TDZD-8 is a non-ATP competitive GSK-3β inhibitor; ③ Kenpaulone (kp) is a glycogen synthase kinase 3 (GSK3); ④ cyclin dependent kinase (CDK) inhibitor. δ-catenin (δ-cat) is an intercellular junction protein, which is a member of the classical catenin family; ⑤ The KCC2 gene, which encodes a molecule known to help expel chloride ions from neurons; ⑥ Mitochondrial reactive oxygen species clusters (mtROS). *The figure was drawn by first author Yong Wang using Biorender software*

**Fig. 2:**
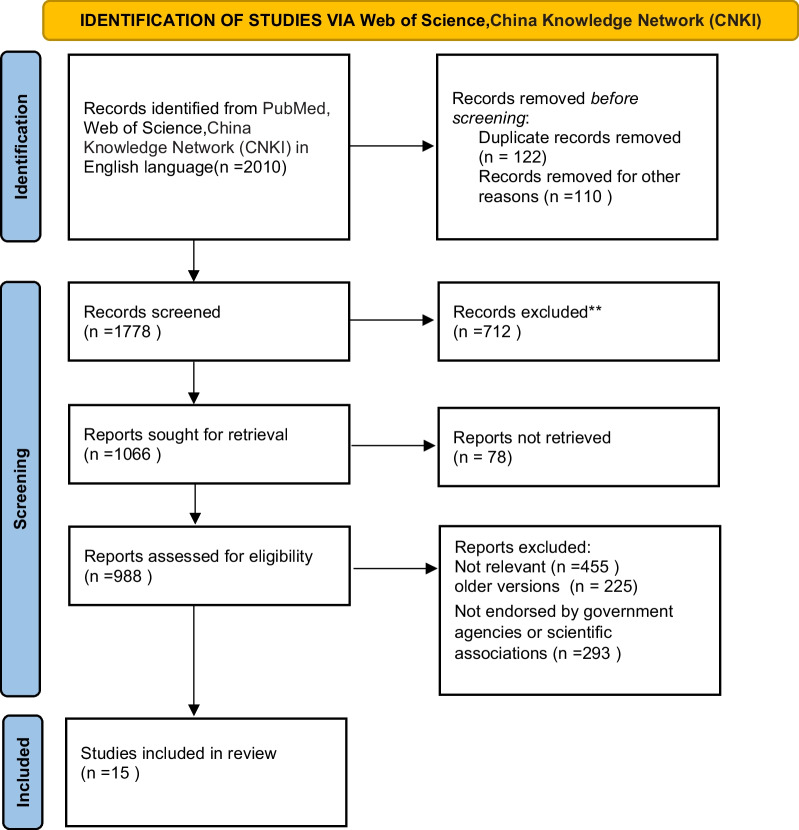
PRISMA flow diagram

Abnormal bone remodeling activates the GSK-3β/Drp1 pathway, thus leading to mitochondrial fission and dysfunction. fd/MAS can activate NLRP3 inflammatory vesicles, which then induce an injurious response. The intrathecal injection of the GSK-3β inhibitor TDZD-8 decreased Drp1 activity, maintained mitochondrial function, reduced the NLRP3 inflammatory vesicle cascade response, and ultimately attenuated FD/MAS pain behavior, as shown in the mechanism in Fig. 1 below (Fig. 2).

## Discussion

Randomized controlled trials are necessary for FD/MAS, especially given the significant placebo effect in clinical pain trials; however, they are difficult to perform, and this represents a gap in the field. Disease modifiers that have previously acted in some way can directly or indirectly affect osteoclast function. Pharmacological treatments using bisphosphonates, denosumab, and surgery also fail to provide sustained pain relief in FD/MAS patients and have significant side effects.

The development of novel mechanism-based therapies may clinically improve the treatment of FD/MAS pain; however, the fundamental improvement of the treatment of FD/MAS pain requires continued research efforts to develop new therapies with multimodal effects to target the underlying mechanisms of different pain conditions. The author believes that the neurochemical and electrophysiological properties of DRG neurons, especially those new nonneuronal and molecular mechanisms, mechanically loaded responsive neurons, and new targets for potential clinical interventions, are future research directions. A large number of animal experiments, tissue engineering techniques, and clinical trials are still needed to verify the effectiveness of the targets in the future.

## Conclusion

In this review, we have focused on collating the clinical research advances in the last 5 years on the mechanisms of FD/MAS pain and the studies that may serve as new pathways in terms of basic experiments. In terms of a complete mechanistic understanding of FD/MAS pain conditions, new research results on new signaling pathways, neurotrophic factor receptors, purinergic receptors, interferon-stimulating factors, potassium channels, protein kinases, and corresponding hormonal modulation are organized, and their respective advantages and disadvantages are summarized separately to make the included articles more updated and complete and to summarize the therapeutic approaches that have been identified by the latest studies. The neuronal activity of FD/MAS patients is at a consistently high level, which leads to an increased sensitivity to painful stimuli. However, the treatment of pain due to FD/MAS also requires a comprehensive consideration of skeletal deformities. This article does not describe in detail the mechanical pain caused by skeletal deformities.

## Abbreviations

ASICs: Acid-sensing ion channels
BDNF: Brain-derived neurotrophic factor
CPG: Central pattern generator
CREBs: Cyclic phospho-adenosine effector-binding proteins
CPG: Central pattern generator
CGRP: Calcitonin gene-related peptide
cGAS: Cyclic GMP-AMP synthase
DRG: Dorsal root ganglia
ES: Estrogen
EPO: Erythropoietin
ERKs: Extracellularly regulated protein kinases
FD: Fibrous dysplasia
GPCRs: G protein-coupled receptors
OGR1: G protein-coupled receptor 1
GSK-3: Glycogen synthase kinase-3
IGF-1: Insulin-like growth factor-1
IL-1β: Interleukin-1β
AN: Lipocalin
MAS: McCune–Albright syndrome
mtROS: Mitochondrial reactive oxygen species clusters
NMDA: N-methyl-D-aspartate
NGF: Nerve growth factor
OS: Oxidative stress
OT: Oxytocin
PTH: Parathyroid hormone
PD: Parkinson’s disease
P2XRs: Purinergic receptors
PAG: Periaqueductal gray
p38-MAPK: P38 protein kinase
TrKA: Promyosin receptor kinase A
ROS: Reactive oxygen species
Runx2: Runt-related transcription Factor 2
SIRT6: Recombinant Sirtuin 6
Sost: Sclerostin
TRP: Transient receptor potential
TRPM7: Transient receptor potential cation channel subfamily M
TRPV: Transient receptor potential channel subfamily V
TBK1: TANK-binding kinase 1
VEGF: Vascular endothelial growth factor
VGICs: Voltage-gated ion channels
YAP: Yes-associated protein

## Declarations

### Related statements

The authors have read the PRISMA 2009 Checklist, and the manuscript was prepared and revised according to the PRISMA 2009 Checklist.
